# Association between carotid plaque calcification and clinical outcomes of symptomatic cerebral small vessel disease

**DOI:** 10.3389/fneur.2025.1628353

**Published:** 2025-08-14

**Authors:** Jing Zhang, Shuo Zhao, Xunyao Hou, Xinyao Wang, Yunliang Guo, Ximing Wang

**Affiliations:** ^1^School of Medicine, Shandong First Medical University, Jinan, China; ^2^Department of Radiology, Shandong Provincial Hospital Affiliated to Shandong First Medical University, Jinan, China; ^3^Department of Radiology, Liaocheng People’s Hospital, Shandong First Medical University, Liaocheng, China; ^4^Department of Radiology, Shandong Provincial Hospital, Cheeloo College of Medicine, Shandong University, Jinan, China; ^5^Department of Geriatric Neurology, Shandong Provincial Hospital Affiliated to Shandong First Medical University, Jinan, China; ^6^Department of Geriatrics, Shandong Provincial Hospital Affiliated to Shandong First Medical University, Jinan, China

**Keywords:** atherosclerosis, calcification, cerebral small vessel disease, clinical outcomes, computed tomography angiography

## Abstract

**Objective:**

Atherosclerosis is the most common pathological change of cerebral small vessel disease (CSVD). This study aimed to investigate correlations between carotid atherosclerotic calcification and clinical outcomes of symptomatic CSVD.

**Methods:**

We retrospectively evaluated 210 symptomatic CSVD patients who underwent carotid computed tomography angiography (CTA) and brain magnetic resonance imaging (MRI). Clinical outcomes were evaluated using modified Rankin Scale (mRS) at 90 days after acute event. The presence and characteristics of carotid calcification (including size, number and location), carotid plaque burden and CSVD markers were analyzed. Logistic regression analysis was used to explore associations between carotid calcification and CSVD outcomes. Key confounding variables (age, sex, hypertension, diabetes mellitus, hyperlipidemia, coronary heart disease, smoking history, drinking history, ulceration, positive remodeling and positive soft plaque) were adjusted in multivariate analysis. The areas under the curve (AUC) of receiver operating characteristic (ROC) curves were used to analyze predictive performance of various radiological variables for CSVD outcomes.

**Results:**

A total of 129 patients with poor outcomes and 81 with good outcomes were analyzed. The incidence of calcification plaque in poor outcome group was higher than those in good outcome group (*p* = 0.001). Logistic regression found that the presence of calcification, surface calcification, multiple calcifications, thick/mixed calcifications, carotid stenosis degree and total CSVD score were associated with adverse outcomes in symptomatic CSVD before and after adjusting for confounding factors (all *p* < 0.05). ROC analysis showed that the prediction model with integrated carotid calcification exhibited enhanced performance with a sensitivity of 75.19% and specificity of 70.37% (AUC = 0.752, *p* < 0.001).

**Conclusion:**

Carotid calcification characteristics were associated with clinical outcomes of symptomatic CSVD, which could be used as predictive indicators of CSVD outcomes.

## 1 Introduction

Cerebral small vessel disease (CSVD) refers to a group of pathological processes affecting the small arteries, venules, and capillaries of the brain (1). With a high prevalence in the elderly, CSVD accounts for approximately one-quarter of all strokes (2). Typical neuroimaging markers of CSVD include recent small subcortical infarct (RSSI), lacune, white matter hyperintensity (WMH), enlarged perivascular space (EPVS), cerebral microbleed (CMB) and brain atrophy (3). Among various subtypes, symptomatic CSVD is a clinical presentation of a lacunar syndrome, confirmed by anatomically corresponding lacunar infarction on brain MRI (4). The clinical manifestation of symptomatic CSVD is complicated, and its clinical outcomes vary substantially among individuals (5). Therefore, early detection of symptomatic CSVD patients with a high risk of developing adverse outcomes is essential.

Atherosclerosis of carotid arteries was a well-established cause of ipsilateral ischemic stroke and was also one of the most common pathological changes of CSVD (1, 6). Arterial calcification, which was an indicator of advanced atherosclerosis (7), has been recognized as an easily identifiable and highly specific imaging feature reflecting atherosclerosis. Historically, calcification has been considered protective and a marker of plaque stability (8, 9). However, several recent studies have emphasized that calcification in specific sizes and locations was associated with increased plaque vulnerability (10, 11), which in turn is related to CSVD (12, 13).

Lots of studies have identified that carotid calcification was an adverse factor for cerebrovascular ischemic events (e.g., stroke, transient ischemic attack) (14–17). However, most of these studies focused on the calcification volume or subtype, and few studies focused on the characteristics of carotid artery calcification. Compared with previous studies, characteristics of carotid artery calcified plaques could comprehensively depict the distribution, shape, stability and other factors of calcification, which may better evaluate the prognosis of patients with cerebrovascular ischemic events. Additionally, prior research has shown that carotid diseases (e.g., carotid vulnerable plaque, carotid stenosis) were associated with poor post-stroke outcomes (18, 19). Nevertheless, it is uncertain whether characteristics of carotid artery calcification are correlated with clinical outcomes of symptomatic CSVD.

Therefore, this study aimed to explore the potential link between carotid atherosclerotic calcification and the clinical outcomes of symptomatic CSVD patients, as well as to determine whether this correlation is dependent on the specific feature of calcification.

## 2 Materials and methods

### 2.1 Study population

**Figure 1:**
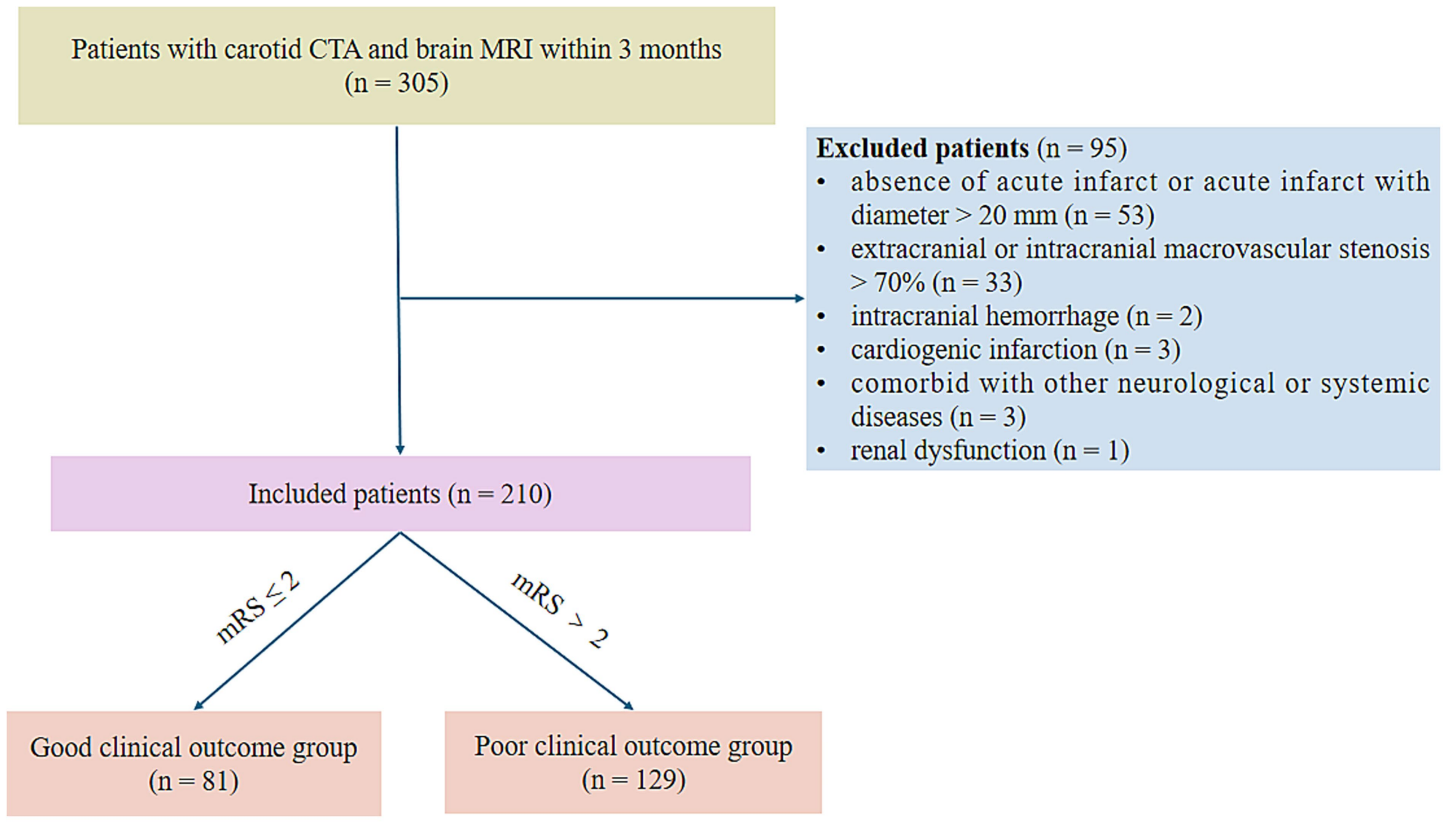
Flow chart for patient selection. CTA, computed tomography angiography; mRS, modified Rankin Scale.

This study was approved by the Ethical Committee of Shandong Provincial Hospital Affiliated to Shandong First Medical University (SWYX: No. 2024–072), and informed consent was waived due to the retrospective nature of the study. We retrospectively analyzed 305 patients diagnosed with symptomatic CSVD defined as a clinical presentation of lacunar syndrome confirmed by anatomically corresponding lacunar infarct on brain MRI from January 2016 to January 2024. Inclusion criteria: (1) MRI-verified symptomatic lacunar ischemic stroke; (2) typical MRI findings of CSVD, including RSSI, lacune, WMH, EPVS, and CMB; (3) carotid CTA and brain MRI were conducted successively in the short term (≤ 3 months). Exclusion criteria were: (1) absence of acute infarct or acute infarct with diameter > 20 mm (*n* = 53); (2) extracranial or intracranial macrovascular stenosis > 70% (*n* = 33); (3) intracranial hemorrhage (*n* = 2); (4) cardiogenic infarction (*n* = 3); (5) WMH unrelated to CSVD, such as multiple sclerosis (*n* = 1); (6) comorbid with other neurological disorders, such as Parkinson’s disease (*n* = 1); (7) comorbid with systemic diseases, such as tumors (*n* = 1). (8) Renal dysfunction (*n* = 1). The patients’ selection flow chart is shown in Figure 1.

Comprehensive data including demographics, clinical features and risk factors were gathered. Patient demographics and clinical data are listed in Table 1. Clinical outcomes were based on the modified Rankin Scale (mRS) score at 90 days after acute event. A good outcome was classified as mRS ≤ 2, while mRS > 2 was considered as a poor outcome.

**Table 1:** Baseline characteristics of the study population.

Variables	Patients (*n* = 210)
Clinical risk factors	
Age, median (IQR)	61 (54–68)
Sex, male, *n* (%)	159 (75.7)
Hypertension, *n* (%)	158 (75.2)
Diabetes mellitus, *n* (%)	66 (31.4)
Hyperlipidemia, *n* (%)	88 (41.9)
Coronary heart disease, *n* (%)	30 (14.3)
Smoking history, *n* (%)	85 (40.5)
Drinking history, *n* (%)	97 (46.2)
Medication, *n* (%)	
Antiplatelet therapy	201 (95.7)
Statins	192 (91.4)
Antihypertensive therapy	137 (65.2)
Antidiabetic therapy	61 (29.0)
Clinical variable	
NIHSS, median (IQR)	3 (2–5)
mRS score at baseline, median (IQR)	3 (3–4)
mRS score at 90 days, median (IQR)	3 (2–3)

mRS, modified Rankin scale; NIHSS, National Institutes of Health Stroke Scale; IQR, interquartile range.

### 2.2 CTA and MRI protocols

Carotid CTA was performed with a third-generation dual-source CT scanner (SOMATOM Force, Siemens Healthcare). Contrast medium (Omnipaque-350, GE Healthcare) was injected at 4 mL/s with a volume of 60–80 mL, followed immediately by 40 mL of saline solution at the same injection rate. Bolus tracking was utilized to start the acquisition 5 s after the aortic arch attenuation reached the 100 HU threshold. The following parameters were set for the examination: Tube voltage, 80 kV for BMI ≤ 25 kg/m^2^ and 100 kV for BMI > 25 kg/m^2^; pitch, 1.0; rotation time, 350 ms; reconstructed slice thickness, 1.5 mm; reconstructed slice interval, 1.0 mm.

The brain MRI examination was performed using a 3.0 Tesla MRI system (Magnetom Prisma, Siemens Healthineers, Erlangen, Germany). The scan sequence included: T1-weighted images (T1WI), T2-weighted images (T2WI), T2 -weighted fluid-attenuated inversion recovery (T2-FLAIR), diffusion-weighted imaging (DWI) and susceptibility- weighted imaging (SWI). The sequence parameters are as follows: T1WI: time repetition (TR) = 2000 ms, time echo (TE) = 7.4 ms, and slice thickness (ST) = 5.0 mm. T2WI: TR/TE = 4,300 ms/109 ms, ST = 5.0 mm; T2-FLAIR: TR/TE = 8,000 ms/81 ms, ST = 5.0 mm; DWI: TR/TE = 2,680 ms/57 ms, ST = 1.5 mm; SWI: TR/TE = 27 ms/20 ms, ST = 1.5 mm.

### 2.3 Imaging assessment

The CTA and MRI images were independently evaluated by two experienced radiologists (JZ and SZ), each with more than 5 years of specialized experience in neurovascular imaging. Any disagreement was referred to a senior radiologist (XW) for a final decision. All image assessments were performed blinded to clinical data.

#### 2.3.1 Carotid artery CTA

**Figure 2:**
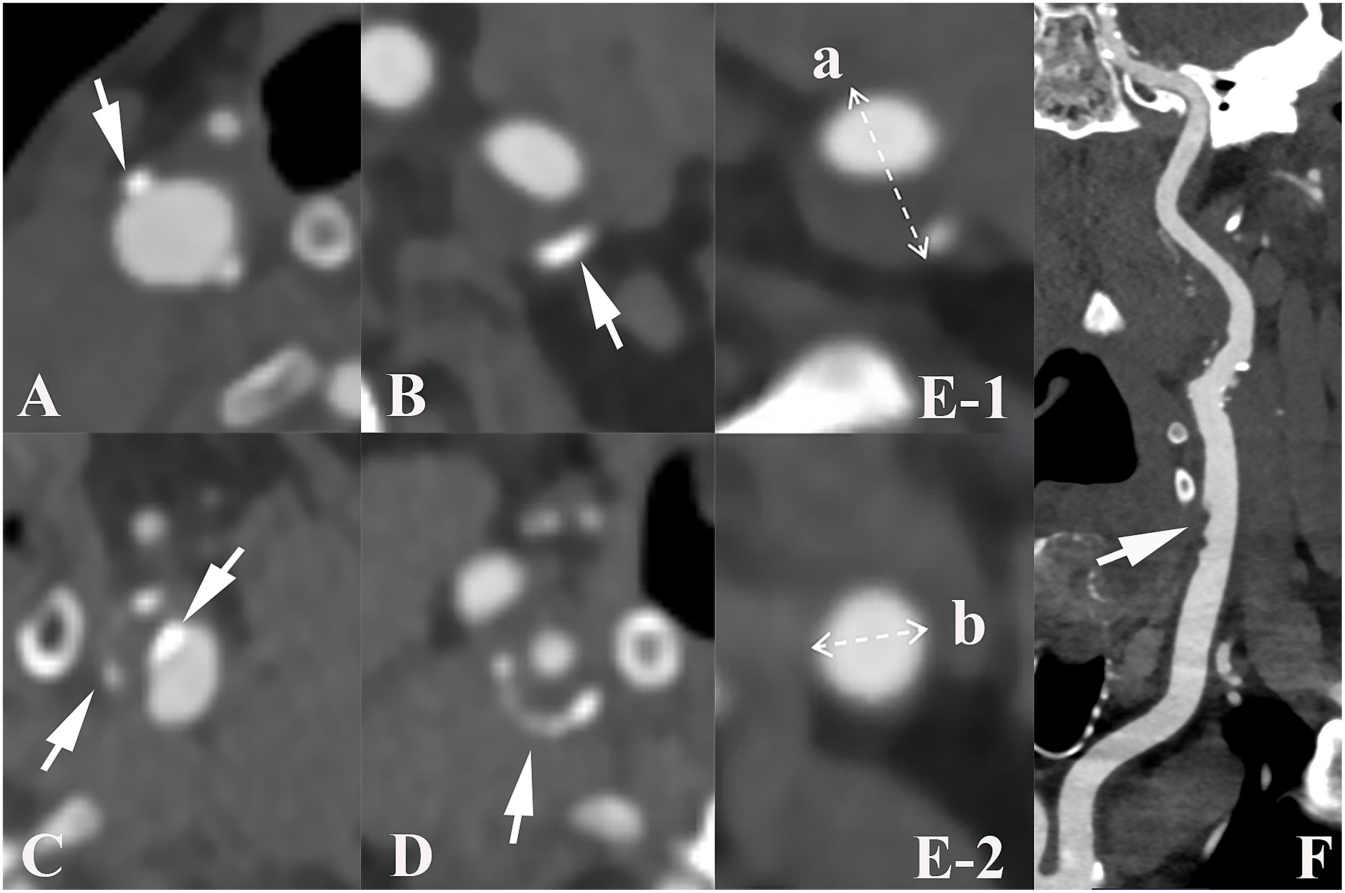
Imaging characteristics of carotid calcified plaque, positive remodeling and ulceration. Arrows show plaque with various characteristics: **(A–C)** illustrate the different positions of calcified plaques in carotid artery. **(A)**, surface calcification; **(B)**, deep calcification; **(C)**, mixed calcification. Panel **(D)** indicates calcification with a positive rim sign. Panel **(E)** presents an example of positive remodeling; remodeling index (RI) was computed as a/b. Panel **(F)** shows ulceration (arrow) of carotid plaque.

Carotid arteries analyzed mainly included common carotid arteries, extracranial internal carotid arteries, and intracranial internal carotid arteries. Using a postprocessing workstation (Syngovia, Siemens Force, Germany) to analyze and record the presence, number, location, maximum thickness and rim sign of calcified plaques on unilateral or bilateral carotid arteries. Depending on the quantity of plaques, calcified plaques were classified as either single (< 2) or multiple (≥ 2). Based on the location of calcified plaques, they were categorized as surface, deep, or mixed (presence of both surface and deep calcification). Surface calcification was identified when it was situated at or adjacent to the intimal surface, whereas deep calcification was defined when it was positioned at or near the medial/adventitial border with complete encasement by fibrous tissue (20). According to the maximum thickness of the calcified plaque, the morphology of calcification was divided into thin (< 2 mm), thick (≥2 mm), and mixed (both thin and thick calcification were present) (21). The rim sign was defined as a thick soft plaque (≥2 mm) surrounded by thin calcium (< 2 mm) (22). Examples of various imaging characteristics of calcification were shown in Figure 2.

Additionally, the degree of carotid artery stenosis, plaque ulceration, plaque remodeling index (RI) and positive soft plaque were recorded. A maximum soft plaque thickness of more than 2 mm was considered positive soft plaque (21). According to the North American Symptomatic Carotid Endarterectomy Trial criteria (23), the degree of carotid artery stenosis is classified as mild (0–49.9%), moderate (50–69.9%), or severe (70–99.9%). The last group was excluded because of severe stenosis. Ulceration was defined as contrast media protrusion into the plaque, measuring at least 2 mm (24). RI was defined as the ratio of the maximum wall thickness at the most stenotic location to the wall thickness at the normal proximal vessel and RI ≥ 1.05 indicates positive remodeling (25).

#### 2.3.2 Assessment of MRI markers of CSVD

Imaging features of CSVD are assessed in accordance with the Standards for Reporting Vascular Changes on Neuroimaging consensus. (1) Lacune, a round or oval structure with a diameter of 3–15 mm located subcortically. The signal of lacune is similar to cerebrospinal fluid on T1WI and T2WI; it exhibits hyperintensity surrounding rims on FLAIR (26). (2) WMH, shown as isointensity or hypointensity on T1WI while displaying hyperintensity on FLAIR and T2WI. It is classified as periventricular WMH (PWMH) and deep WMH (DWMH) according to the Fazekas scale and graded from 0 to 3 (27). (3) CMB, a small round or ovoid low signal on SWI sequences, with a diameter of 2 to 10 mm (28). (4) EPVS, presented as round or linear cerebrospinal fluid signals on T1WI, T2WI and FLAIR sequences with a diameter of < 3 mm. A validated four-point visual assessment scale (score 0: none; score 1: 1–10; score 2: 11–20; score 3: 21–40; score 4: > 40) was used to assess the EPVS of the basal ganglia (29). The overall CSVD score was assessed by utilizing an established scale ranging from 0 to 4 (30). The presence of the manifestations listed below earned one point: (1) ≥ 1 lacuna; (2) Fazekas score with deep WMH ≥ 2 points and/or periventricular WMH score of 3 points; (3) ≥ 1 deep or infratentorial CMB; (4) severe PVS in the basal ganglia (≥ 2 points).

### 2.4 Statistical analysis

The Kolmogorov–Smirnov test was used to determine the variable’s normality. Continuous quantitative data was presented as mean ± standard deviation (SD) or median [interquartile range, IQR] based on distribution, and Student’s t-test or Mann–Whitney *U* test were used for comparison. Categorical variables were described as frequencies (percentages), and intergroup differences are compared using the *χ*^2^ test or Fisher’s exact test. Univariate and multivariate logistic regressions were used to explore associations between calcification characteristics, plaque burden, CSVD markers and outcomes in symptomatic CSVD patients. In multivariate analysis, commom clinical risk factors and carotid plauqe burden were adjusted. Interaction terms were used to investigate whether the association between carotid calcification and outcomes of symptomatic CSVD differed according to clinical covariates. The areas under the curve (AUC) of the receiver operating characteristic (ROC) curves were reported to evaluate the possible incremental contribution of carotid calcification features (*p* value *<* 0.05 in multivariate regression analysis) to identify poor outcomes. Bootstrap method was used to internally validate models with 500 resamples (31). Calibration curves and Decision curve analysis (DCA) were used to evaluate the calibration ability and net benefits of models. Two-sided *p* values *<* 0.05 were considered to be statistically significant. The interobserver agreements for CTA data and CSVD markers were assessed using Kappa test, with values greater than 0.75 considered excellent reproducibility. All statistical analyses were conducted using SPSS software (IBM, Inc., version 26.0).

## 3 Results

### 3.1 Baseline characteristics

A total of 210 patients (median age 61 years, IQR 54–68) were enrolled; 159 (75.7%) were men; 158 (75.2%) had hypertension, 66 (31.4%) had diabetes mellitus, 88 (41.9%) had hyperlipidemia and 30 (14.3%) had coronary heart disease. More baseline characteristics are presented in Table 1.

The distribution of mRS scores at baseline and at 90 days were shown in Supplementary Figure S1. As shown in Table 2, 129 patients were identified as having poor outcomes 90 days after acute event and 81 had good outcomes. Compared with patients without good outcomes, those with poor outcomes were more likely to be older (*p* = 0.015) and to have higher NIHSS scores at admission (*p <* 0.001). No significant difference was found in other clinical risk factors between two groups (all *p* > 0.05).

**Table 2:** Baseline characteristics between groups with good and poor outcomes.

Variables	Good outcome group (mRS ≤ 2, *n* = 81)	Poor outcome group (mRS > 2, *n* = 129)	*p* value
Clinical risk factors			
Age (≥ 60), *n* (%)	37 (45.7)	81 (62.8)	**0.015** ^*^
Sex, male, *n* (%)	56 (69.1)	103 (79.8)	0.078
Hypertension, *n* (%)	56 (69.1)	102 (79.1)	0.104
Diabetes mellitus, *n* (%)	22 (27.2)	44 (34.1)	0.291
Hyperlipidemia, *n* (%)	38 (46.9)	50 (38.8)	0.244
Coronary heart disease, *n* (%)	7 (8.6)	23 (17.8)	0.064
Smoking history, *n* (%)	29 (35.8)	56 (43.4)	0.274
Drinking history, *n* (%)	39 (48.1)	58 (45.0)	0.652
Medication, *n* (%)			
Antiplatelet therapy	78 (96.3)	123 (95.3)	0.741
Statins	71 (87.7)	121 (93.8)	0.122
Antihypertensive therapy	52 (64.2)	85 (65.9)	0.802
Antidiabetic therapy	18 (22.2)	43 (33.3)	0.084
Clinical variable			
NIHSS, median (IQR)	1 (1–2)	5 (3–6)	**<0.001** ^***^

Bold values indicate statistical significance. ^*^p < 0.05, ^***^p < 0.001.

mRS, modified Rankin scale; NIHSS, National Institutes of Health Stroke Scale; IQR, interquartile range.

### 3.2 Comprision of imaging characteristics

Imaging parameters were compared between two groups and exhibited in Table 3. Patients with poor outcomes tended to develop lacune (*p* < 0.001), severe periventricular and deep WMH (*p*s < 0.001), CMB (*p* = 0.003) and higher total CSVD burden (*p* < 0.001). As for plaque burden, severe carotid stenosis (*p* < 0.001) and positive remodeling (*p* = 0.031) were frequently observed in poor outcome group. Moreover, the incidence of calcification (*p* = 0.001), surface calcification (*p* = 0.001), multiple calcifications (*p* < 0.001) and thick/mixed calcifications (*p* < 0.001) were significantly higher in patients with adverse outcomes compared to those with good outcomes. However, ulceration, the incidence of positive soft plaque, deep/mixed calcifications, single calcification, thin calcification and rim sign showed no significant difference between two groups (all *p* > 0.05).

**Table 3:** Comparisons of CSVD markers, plaque burden and calcification.

Variables	Good outcome group (mRS ≤ 2, *n* = 81)	Poor outcome group (mRS > 2, *n* = 129)	*p* value
CSVD markers			
Lacunes, *n* (%)	29(35.8)	92(71.3)	**< 0.001** ^***^
Periventricular WMH, *n* (%)			**< 0.001** ^***^
Grade 0	1 (1.2)	0 (0.0)	
Grade 1	17 (21.0)	8 (6.2)	
Grade 2	45 (55.6)	63 (48.8)	
Grade 3	18 (22.2)	58 (45.0)	
Deep WMH, *n* (%)			**< 0.001** ^***^
Grade 0	9 (11.1)	0 (0.0)	
Grade 1	20 (24.7)	18 (14.0)	
Grade 2	42 (51.9)	69 (53.5)	
Grade 3	10 (12.3)	42 (32.6)	
EPVS, median (IQR)	2 (2–3)	2 (2–3)	0.434
CMB, *n* (%)	4 (4.9)	25 (19.4)	**0.003** ^**^
Total CSVD score, median (IQR)	2 (1–3)	3 (2–3)	**< 0.001** ^***^
Plaque burden, *n* (%)			
Stenosis degree			**< 0.001** ^***^
None	18(22.2)	10(7.8)	
< 30%	62 (76.5)	77 (59.7)	
30–69%	1 (1.2)	42 (32.6)	
Ulceration	2 (2.5)	2 (1.6)	0.640
Positive remodeling	1 (1.2)	11 (8.5)	**0.031** ^*^
Positive soft plaque	24 (29.6)	47 (36.4)	0.310
Calcification, *n* (%)			
Presence	62 (76.5)	119 (92.2)	**0.001** ^**^
Location			
Surface	40 (49.4)	93 (72.1)	**0.001** ^**^
Deep/mixed	22 (27.2)	27 (20.9)	0.299
Number			
Single	17 (21.0)	18 (14.0)	0.183
Multiple	45 (55.6)	101 (78.3)	**< 0.001** ^***^
Size			
Thin	30 (37.0)	33 (25.6)	0.078
Thick/mixed	32 (39.5)	86 (66.7)	**< 0.001** ^***^
Rim sign (+)	3 (3.7)	10 (7.8)	0.236

Bold values indicate statistical significance. ^*^p < 0.05, ^**^p < 0.01, ^***^p < 0.001.

CMB, cerebral microbleed; CSVD, cerebral small vessel disease; EPVS, enlarged perivascular space; mRS, modified Rankin scale; WMH, white matter hyperintensity. IQR, interquartile range.

Besides, excellent inter-reader agreements were obtained in CSVD markers and CTA variables as well (lacune: *k* = 0.875; periventricular WMH: *k* = 0.795; deep WMH: *k* = 0.836; EPVS: *k* = 0.894; CMB: *k* = 0.857; CSVD total score: *k* = 0.840; carotid stenosis: *k* = 0.864; ulceration: *k* = 0.773; positive remodeling: *k* = 0.773; positive soft plaque: *k* = 0.886; presence of calcification: *k* = 1.000; surface calcification: *k* = 0.800; deep/mixed calcification: *k* = 0.802; single calcification: *k* = 0.828; multiple calcificationsrim sign; thin calcification: *k* = 0.765; thick/mixed calcifications: *k* = 0.783; rim sign: *k* = 0.828).

### 3.3 Logistic regression analyses for poor clinical outcomes

**Figure 3:**
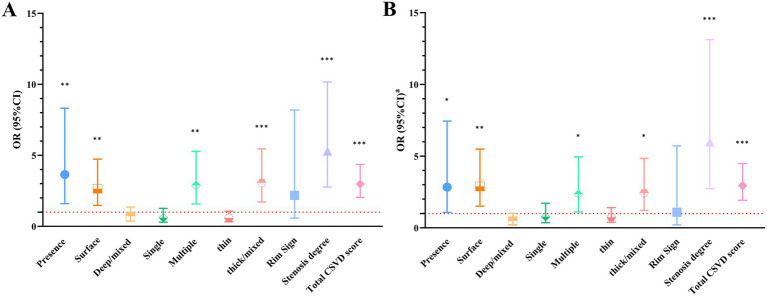
**(A)** Univariable logistic regression results. **(B)** Multivariate logistic regression results. ^a^The results were adjusted for age, sex, hypertension, diabetes mellitus, hyperlipidemia, coronary heart disease, smoking history, drinking history and carotid plaque characteristics (for ulceration, positive remodeling and positive soft plaque). ^*^p < 0.05, ^**^p < 0.01, ^***^p < 0.001. CI, confidence interval; CSVD, cerebral small vessel disease; OR, odds ratio.

In univariate logistic regression analyses, clinical risk factors did not significantly modify the associations between carotid calification features and clinical oucomes (all interaction *p* > 0.05) (Supplementary Table S1); the presence of calcification [odds ratio (OR) = 3.647; 95% CI, 1.598–8.321], surface calcification (OR = 2.648; 95% CI, 1.481–4.735), multiple calcifications (OR = 2.886; 95% CI, 1.574–5.290), thick/mixed calcifications (OR = 3.062; 95% CI, 1.721–5.451), total CSVD score (OR = 2.983; 95% CI, 2.042–4.359) and carotid stenosis degree (OR = 5.300; 95% CI, 2.763–10.166) all showed significant associations with poor outcomes in symptomatic CSVD (Table 4; Figure 3A). After adjusting for sex, age, hypertension, diabetes mellitus, hyperlipidemia, coronary heart disease, smoking history, drinking history and carotid plaque burden (for ulceration, positive remodeling and positive soft plaque), the associations remained (all *p* < 0.05) (Table 4; Figure 3B).

**Table 4:** Association between imaging characteristics and outcomes of symptomatic CSVD.

Variables	Univariate OR (95% CI)	*p* value	Multivariate OR (95% CI)^a^	*p* value
Presence	3.647 (1.598–8.321)	**0.002** ^**^	2.832 (1.078–7.442)	**0.035** ^*^
Location				
Surface	2.648 (1.481–4.735)	**0.001** ^**^	2.884 (1.513–5.495)	**0.001** ^**^
Deep/mixed	0.710 (0.371–1.357)	0.300	0.473 (0.219–1.021)	0.057
Number				
Single	0.610 (0.294–1.268)	0.186	0.785 (0.359–1.716)	0.544
Mutiple/mixed	2.886 (1.574–5.290)	**0.001** ^**^	2.343 (1.110–4.945)	**0.026** ^*^
Size				
Thin	0.584 (0.321–1.065)	0.079	0.736 (0.387–1.401)	0.351
Thick/mixed	3.062 (1.721–5.451)	**<0.001** ^***^	2.427 (1.216–4.846)	**0.012** ^*^
Rim sign	2.185 (0.583–8.191)	0.246	1.083 (0.206–5.708)	0.925
Stenosis degree	5.300 (2.763–10.166)	**<0.001** ^***^	5.988 (2.732–13.123)	**<0.001** ^***^
Total CSVD score	2.983 (2.042–4.359)	**<0.001** ^***^	2.936 (1.926–4.478)	**<0.001** ^***^

Bold values indicate statistical significance. ^*^p < 0.05, ^**^p < 0.01, ^***^p < 0.001.

^a^The results were adjusted for age, sex, hypertension, diabetes mellitus, hyperlipidemia, coronary heart disease, smoking history, drinking history and carotid plaque characteristics (for ulceration, positive remodeling and positive soft plaque).

CSVD, cerebral small vessel disease; OR, odds ratio; CI, confidence interval.

### 3.4 Predictive value of carotid calcification characteristics in clinical outcome

**Figure 4:**
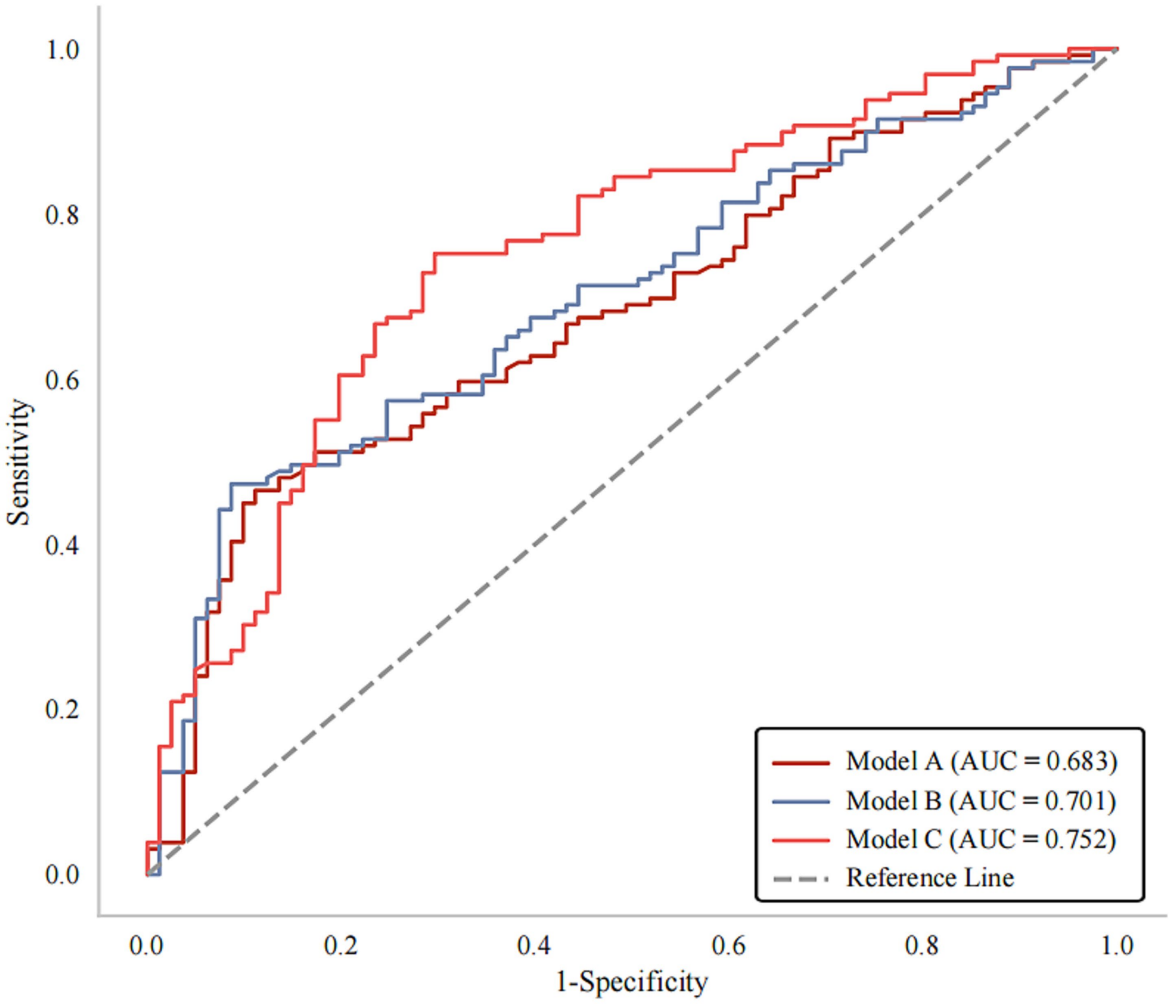
ROC curves for prediction of poor outcomes at 90 days after stroke. Model A: Clinical variables; Model B: Clinical variables + non-calcified plaque features; Model C: Clinical variables + non-calcified + calcified plaque features. AUC, areas under the curve; ROC, receiver operating characteristics.

As shown in Figure 4, three prediction models including variables of clinical covariates, non-calcified plaque features and calcified plaque features were constructed. The AUC for Model A and Model B was 0.683 (*p* < 0.001) and 0.701 (*p* < 0.001) respectively. When adding calcification into Model B, the prediction model (Model C) reached an AUC of 0.752 (95% CI, 0.684–0.820, *p* < 0.001), with a bootstrap-corrected AUC of 0.752 (95% CI, 0.681–0.819, *p* < 0.001) (Supplementary Figure S2). Moreover, Model C had higher sensitivity (75.19% vs. 47.29%) but lower specificity (70.37% vs. 91.36%) compared to Model B (Table 5). As shown in Supplementary Figure S3, Model C exhibited good consistency between predicted and actual risks. Moreover, DCA curves suggested that Model C could provide better clinical benefits than other models (Supplementary Figure S4).

**Table 5:** Influence of variables on predictive accuracy of models.

Models	Included variables	AUC (95% CI)	*p* value	Sensitivity (%)	Specificity (%)
A	Clinical variables	0.683 (0.611–0.755)	**< 0.001** ^***^	46.51	87.65
B	Clinical variables + non-calcified plaque features	0.701 (0.631 ~ 0.772)	**< 0.001** ^***^	47.29	91.36
C	Clinical variables + non-calcified + calcified plaque features	0.752 (0.684–0.820)	**< 0.001** ^***^	75.19	70.37

Bold values indicate statistical significance. ^***^p < 0.001.

Model A: Clinical variables (age, sex, hypertension, diabetes mellitus, hyperlipidemia, coronary heart disease, smoking history, drinking history); Model B: Clinical variables + non-calcified plaque features (ulceration, positive remodeling, positive soft plaque); Model C: Clinical variables + non-calcified + calcified plaque features (presence of calcification, surface, mutiple and thick/mixed calcifications).

AUC, areas under the curve; CI, confidence interval; CSVD, cerebral small vessel disease.

## 4 Discussion

In this study, we observed that the presence of calcification, specifically surface, multiple and thick/mixed calcifications, was associated with clinical outcomes of symptomatic CSVD before and after adjusting for confounding factors. It is suggested that the presence and features of carotid atherosclerotic calcification may be independent risk factors for predicting poor outcomes of symptomatic CSVD.

Atherosclerosis was intrinsically linked to alterations in the microvasculature, which contributed to CSVD development (32, 33). Carotid artery calcification, as an important marker of large artery atherosclerosis, could lead to arterial stiffness, reduced arterial compliance, increased pulse pressure and perfusion pressure in downstream small cerebral vessels. This resulted in damage to the cerebral microcirculation (20, 34–36). Additionally, atherosclerotic calcification was considered as a risk factor associated with plaque instability (37); vascular inflammation was prominent during the early stage of calcification (38) and could also contribute to plaque instability. The aforementioned pathophysiological changes may potentially lead to the formation of microemboli (39), thereby causing the occurrence of brain parenchymal injury, which may contribute to adverse outcomes of symptomatic CSVD.

Our findings indicated that individuals with surface, multiple and thick/mixed calcifications had a higher likelihood of experiencing poor outcomes. Although relationships between carotid calcification and CSVD markers were gradually established (40, 41), the mechanism underlying associations between characteristics of calcification and clinical outcomes of CSVD was still unclear. Prior research has indicated that the location, amount and size of calcification can influence the mechanical stress of plaque and alter the stability of the cap, potentially shedding light on this connection (42). Specifically, surface calcification will enhance the risk of neovascular rupture and thrombosis by prominently increasing plaque surface stress (43–45). In contrast, deep/mixed calcification has a negligible impact on plaque maximum stress (21, 46).

Multiple calcified plaques exhibit similar mechanisms. Compared with the soft compositions, the region of calcification showed a lower strain rate. This stress imbalance may lead to rupture of neovascularization at the interface between calcification and soft components. In contrast to single calcification, multiple calcifications may increase the interface surface area, thereby potentially elevating the risk of neovessel rupture (46). Additionally, several studies have demonstrated that surface, multiple and thick calcifications are independent risk predictors of intraplaque hemorrhage (21, 47, 48). These calcification features may reflect plaque vulnerability. Vulnerable plaques may cause oxidative stress, inflammation and microembolic dislodgement, leading to cerebral infarction and functional impairment (12, 19). To some extent, these studies support our observation that surface, multiple and thick calcification is an important determinant of plaque vulnerability, which could lead to adverse outcomes for symptomatic CSVD.

In addition to characteristics of calcification plaques, we found that total CSVD load and degree of carotid stenosis could also predict clinical outcomes of symptomatic CSVD. This finding is consistent with prior studies (49–51). These associations can be explained through several underlying mechanisms. Firstly, when individual neuroimaging markers are taken into account, total CSVD burden can dependably represent the extent of brain tissue affected by ischemia or other forms of injury. Chronic ischemia may lead to stroke, gait impairment, and dementia, which can be attributed to a reduced neural reserve capacity. Secondly, carotid artery stenosis may lead to micro infarctions via artery-to-artery embolization (33). When blood flows through the narrow arteries, it could generate turbulence and low vascular wall shear stress (52). These changes may facilitate the formation of microthrombi and subsequent embolism in small perforating arteries, thereby increasing the risk of cerebrovascular accidents and contributing to adverse outcomes.

We observed that Model C exhibited higher AUC (0.752) and sensitivity (75.19%) than Model B, suggesting that adding calcification into model might possess higher predictive performance and lower rates of missed diagnosis.

Moreover, Moldel C achieved good calibration ability and clinical applicability. This study indicates the potential of utilizing carotid calcification features as clinically practical tools for risk stratification of symptomatic CSVD.

In clinical practice, patients with carotid calcification may require intensified anti-atherosclerotic treatment. Strategies including lifestyle modifications (e.g., quitting smoking, limiting alcohol intake, and engaging in regular moderate exercise), stricter management of blood pressure, glucose, and blood lipid level as well as the use of medications to protect blood vessels, should be implemented to improve prognosis.

This study also has a few restrictions. Firstly, this is a retrospective study and thus only functional outcomes were collected. It is widely recognized that CSVD is a primary cause of vascular cognitive impairment and dementia (53). Therefore, future prospective studies with cognitive outcomes are necessary. Secondly, an independent external validation cohort was not conducted due to single-center nature of current study. Internal validity was assessed through bootstrap resampling methods. Thirdly, the present study did not comprehensively consider mRS score variations during 90 days after stroke. Categorizing CSVD patients based on mRS score changes (deterioration vs. improvement) would be an innovative method. Finally, it should be noted that the clinical outcomes were only followed up 90 days after stroke, and long-term outcomes (e.g., 1 year) need to be further validated.

In conclusion, carotid calcification was observed to be correlated with clinical outcomes of symptomatic CSVD. This association was determined by the location, quantity, and size of calcification. Evaluation of carotid artery calcification may provide a strategy for clinical prevention and treatment of symptomatic CSVD patients.

## Data Availability

The original contributions presented in the study are included in the article/Supplementary material, further inquiries can be directed to the corresponding authors.

## Supplementary material

SUPPLEMENTARY FIGURE S1 The distribution of mRS scores at baseline and at 90 days. mRS, 640 modified Rankin scale.

SUPPLEMENTARY FIGURE S2 ROC curves for bootstrap internal validation. AUC, areas under the curve; CI, confidence interval; ROC, receiver operating characteristics.

SUPPLEMENTARY FIGURE S3 Calibration curves for multiple models. **(A–C)** presented calibration curves of Model **A–C** respectively.

SUPPLEMENTARY FIGURE S4 Decision curves for multiple models.

## References

[1] Pantoni L. Cerebral small vessel disease: from pathogenesis and clinical characteristics to therapeutic challenges. Lancet Neurol. (2010) 9:689–701. doi: 10.1016/s1474-4422(10)70104-6 20610345

[2] Low A Mak E Rowe JB Markus HS O'brien JT. Inflammation and cerebral small vessel disease: a systematic review. Ageing Res Rev. (2019):100916. doi: 10.1016/j.arr.2019.100916 31181331

[3] Wardlaw JM Smith EE Biessels GJ Cordonnier C Fazekas F Frayne R. Neuroimaging standards for research into small vessel disease and its contribution to ageing and neurodegeneration. Lancet Neurol. (2013) 12:822–38. doi: 10.1016/s1474-4422(13)70124-8 23867200 PMC3714437

[4] Nannoni S Ohlmeier L Brown RB Morris RG Mackinnon AD Markus HS. Cognitive impact of cerebral microbleeds in patients with symptomatic small vessel disease. Int J Stroke. (2022) 17:415–24. doi: 10.1177/17474930211012837 33877017

[5] Feng X Taiwakuli M Du J Zhu W Xu S. Clinical and imaging risk factors for early neurological deterioration and long-term neurological disability in patients with single subcortical small infarction. BMC Neurol. (2025) 25:66. doi: 10.1186/s12883-025-04067-x 39955506 PMC11829468

[6] Subedi D Zishan US Chappell F Gregoriades ML Sudlow C Sellar R. Intracranial carotid calcification on cranial computed tomography: visual scoring methods, semiautomated scores, and volume measurements in patients with stroke. Stroke. (2015) 46:2504–9. doi: 10.1161/strokeaha.115.009716 26251250 PMC4542564

[7] Shioi A Ikari Y. Plaque calcification during atherosclerosis progression and regression. J Atheroscler Thromb. (2018) 25:294–303. doi: 10.5551/jat.Rv17020 29238011 PMC5906181

[8] Huang H Virmani R Younis H Burke AP Kamm RD Lee RT. The impact of calcification on the biomechanical stability of atherosclerotic plaques. Circulation. (2001) 103:1051–6. doi: 10.1161/01.cir.103.8.1051 11222465

[9] Nandalur KR Baskurt E Hagspiel KD Phillips CD Kramer CM. Calcified carotid atherosclerotic plaque is associated less with ischemic symptoms than is noncalcified plaque on Mdct. AJR Am J Roentgenol. (2005) 184:295–8. doi: 10.2214/ajr.184.1.01840295 15615991 PMC2955331

[10] Hutcheson JD Maldonado N Aikawa E. Small entities with large impact: microcalcifications and atherosclerotic plaque vulnerability. Curr Opin Lipidol. (2014) 25:327–32. doi: 10.1097/mol.0000000000000105 25188916 PMC4166045

[11] Phyo WSY Shirakawa M Yamada K Kuwahara S Yoshimura S. Characteristics of calcification and their association with carotid plaque vulnerability. World Neurosurg. (2022) 167:e1017–24. doi: 10.1016/j.wneu.2022.08.127 36058484

[12] Li J Tian Y Shi Y Cui Y Lian J Liu P. Association of vulnerable plaques with white matter hyperintensities on high-resolution magnetic resonance imaging. Quant Imaging Med Surg. (2024) 14:3606–18. doi: 10.21037/qims-23-1856 38720851 PMC11074730

[13] Li J Wu H Hang H Sun B Zhao H Chen Z. Carotid vulnerable plaque coexisting with cerebral small vessel disease and acute ischemic stroke: a Chinese atherosclerosis risk evaluation study. Eur Radiol. (2022) 32:6080–9. doi: 10.1007/s00330-022-08757-9 35364716

[14] Bos D Ikram MA Elias-Smale SE Krestin GP Hofman A Witteman JC. Calcification in major vessel beds relates to vascular brain disease. Arterioscler Thromb Vasc Biol. (2011) 31:2331–7. doi: 10.1161/atvbaha.111.232728 21868705

[15] Bos D Portegies ML Van Der Lugt A Bos MJ Koudstaal PJ Hofman A. Intracranial carotid artery atherosclerosis and the risk of stroke in whites: the Rotterdam study. JAMA Neurol. (2014) 71:405–11. doi: 10.1001/jamaneurol.2013.6223 24535643

[16] Gocmen R Arsava EM Oguz KK Topcuoglu MA. Atherosclerotic intracranial internal carotid artery calcification and intravenous thrombolytic therapy for acute ischemic stroke. Atherosclerosis. (2018) 270:89–94. doi: 10.1016/j.atherosclerosis.2018.01.035 29407893

[17] Nandalur KR Baskurt E Hagspiel KD Finch M Phillips CD Bollampally SR. Carotid artery calcification on Ct may independently predict stroke risk. AJR Am J Roentgenol. (2006) 186:547–52. doi: 10.2214/ajr.04.1216 16423966 PMC2955288

[18] Badacz R Kabłak-Ziembicka A Urbańczyk-Zawadzka M Banyś RP Musiałek P OdrowąŻ-PieniąŻek P. Magnetic resonance imaging and clinical outcome in patients with symptomatic carotid artery stenosis after carotid artery revascularization. Postepy Kardiol Interwencyjnej. (2017) 3:225–32. doi: 10.5114/aic.2017.70190 PMC5644041 29056995

[19] Cheng L Zheng S Zhang J Wang F Liu X Zhang L. Multimodal ultrasound-based carotid plaque risk biomarkers predict poor functional outcome in patients with ischemic stroke or Tia. BMC Neurol. (2023) 23:13. doi: 10.1186/s12883-023-03052-6 36631804 PMC9835263

[20] Wang Y Li C Ding M Lin L Li P Wang Y. Carotid atherosclerotic calcification characteristics relate to post-stroke cognitive impairment. Front Aging Neurosci. (2021) 13:682908. doi: 10.3389/fnagi.2021.682908 34113247 PMC8185032

[21] Yang J Pan X Zhang B Yan Y Huang Y Woolf AK. Superficial and multiple calcifications and ulceration associate with intraplaque hemorrhage in the carotid atherosclerotic plaque. Eur Radiol. (2018) 28:4968–77. doi: 10.1007/s00330-018-5535-7 29876705 PMC6223859

[22] Saba L Chen H Cau R Rubeis GD Zhu G Pisu F. Impact analysis of different Ct configurations of carotid artery plaque calcifications on cerebrovascular events. AJNR Am J Neuroradiol. (2022) 43:272–9. doi: 10.3174/ajnr.A7401 35121588 PMC8985662

[23] North American Symptomatic Carotid Endarterectomy Trial. Methods, patient characteristics, and progress. Stroke. (1991) 22:711–20. doi: 10.1161/01.str.22.6.711 2057968

[24] Jm UK-I Fox AJ Aviv RI Howard P Yeung R Moody AR. Characterization of carotid plaque hemorrhage: a CT angiography and MR intraplaque hemorrhage study. Stroke. (2010) 41:1623–9. doi: 10.1161/strokeaha.110.579474 20576955

[25] Mei Y Yu S Li Z Chen H Zhang J Tan S. Plaque characteristics associated with failure of primary balloon angioplasty for intracranial atherosclerotic stenosis: a retrospective study. J Neurointerv Surg. (2024) 17:99–106. doi: 10.1136/jnis-2023-021295 38296609

[26] Hong YJ Kim CM Kim JE Roh JH Kim JS Seo SW. Regional amyloid burden and lacune in pure subcortical vascular cognitive impairment. Neurobiol Aging. (2017) 55:20–6. doi: 10.1016/j.neurobiolaging.2017.03.010 28395177

[27] Brauner R Gory B Lapergue B Sibon I Richard S Kyheng M. Effect of small vessel disease severity on blood pressure management after endovascular therapy in the Bp target trial. Eur J Neurol. (2023) 30:1676–85. doi: 10.1111/ene.15759 36852526

[28] Igarashi S Ando T Takahashi T Yoshida J Kobayashi M Yoshida K. Development of cerebral microbleeds in patients with cerebral hyperperfusion following carotid endarterectomy and its relation to postoperative cognitive decline. J Neurosurg. (2021) 135:1122–8. doi: 10.3171/2020.7.Jns202353 33386017

[29] Wen C Gan JH Liu S Lu H Wang LC Wu H. Enlarged perivascular spaces correlate with blood-brain barrier leakage and cognitive impairment in Alzheimer's disease. J Alzheimer's Dis. (2025) 104:13872877251317220. doi: 10.1177/13872877251317220 39924914

[30] Lin MP Brott TG Liebeskind DS Meschia JF Sam K Gottesman RF. Collateral recruitment is impaired by cerebral small vessel disease. Stroke. (2020) 51:1404–10. doi: 10.1161/strokeaha.119.027661 32248770

[31] Wang R Huang K Ying H Duan J Feng Q Zhang X. Serum creatinine to cystatin C ratio in relation to heart failure with preserved ejection fraction. BMC Cardiovasc Disord. (2024) 24:721. doi: 10.1186/s12872-024-04359-z 39702099 PMC11660665

[32] Nyúl-Tóth Á Patai R Csiszar A Ungvari A Gulej R Mukli P. Linking peripheral atherosclerosis to blood-brain barrier disruption: elucidating its role as a manifestation of cerebral small vessel disease in vascular cognitive impairment. Geroscience. (2024) 46:6511–36. doi: 10.1007/s11357-024-01194-0 38831182 PMC11494622

[33] Wang Y Cai X Li H Jin A Jiang L Chen W. Association of intracranial atherosclerosis with cerebral small vessel disease in a community-based population. Eur J Neurol. (2023) 30:2700–12. doi: 10.1111/ene.15908 37294661

[34] Scuteri A Nilsson PM Tzourio C Redon J Laurent S. Microvascular brain damage with aging and hypertension: pathophysiological consideration and clinical implications. J Hypertens. (2011) 29:1469–77. doi: 10.1097/Hjh.0b013e328347cc17 21577138

[35] Tarumi T Ayaz Khan M Liu J Tseng BY Parker R Riley J. Cerebral hemodynamics in normal aging: central artery stiffness, wave reflection, and pressure pulsatility. J Cereb Blood Flow Metab. (2014) 34:971–8. doi: 10.1038/jcbfm.2014.44 24643081 PMC4050241

[36] Tsao CW Seshadri S Beiser AS Westwood AJ Decarli C Au R. Relations of arterial stiffness and endothelial function to brain aging in the community. Neurology. (2013) 81:984–91. doi: 10.1212/Wnl.0b013e3182a43e1c 23935179 PMC3888200

[37] Saba L Nardi V Cau R Gupta A Kamel H Suri JS. Carotid artery plaque calcifications: lessons from histopathology to diagnostic imaging. Stroke. (2022) 53:290–7. doi: 10.1161/strokeaha.121.035692 34753301

[38] Fujimoto D Kinoshita D Suzuki K Niida T Yuki H Mcnulty I. Relationship between calcified plaque burden, vascular inflammation, and plaque vulnerability in patients with coronary atherosclerosis. JACC Cardiovasc Imaging. (2024) 17:1214–24. doi: 10.1016/j.jcmg.2024.07.013 39243232

[39] Picano E Paterni M. Ultrasound tissue characterization of vulnerable atherosclerotic plaque. Int J Mol Sci. (2015) 16:10121–33. doi: 10.3390/ijms160510121 25950760 PMC4463636

[40] Boulouis G Charidimou A Auriel E Haley KE Van Etten ES Fotiadis P. Intracranial atherosclerosis and cerebral small vessel disease in intracerebral hemorrhage patients. J Neurol Sci. (2016) 369:324–9. doi: 10.1016/j.jns.2016.08.049 27653918

[41] Vinke EJ Yilmaz P Van Der Toorn JE Fakhry R Frenzen K Dubost F. Intracranial arteriosclerosis is related to cerebral small vessel disease: a prospective cohort study. Neurobiol Aging. (2021) 105:16–24. doi: 10.1016/j.neurobiolaging.2021.04.005 34004492

[42] Cardoso L Kelly-Arnold A Maldonado N Laudier D Weinbaum S. Effect of tissue properties, shape and orientation of microcalcifications on vulnerable cap stability using different hyperelastic constitutive models. J Biomech. (2014) 47:870–7. doi: 10.1016/j.jbiomech.2014.01.010 24503048 PMC4019736

[43] Gonzálvez-García A Hernández-Matamoros H Jiménez-Valero S Jurado-Román A Galeote G Moreno R. Coronary thrombosis from superficial calcific sheet. J Invasive Cardiol. (2020) 32:E266. doi: 10.25270/jic/19.00501 32999102

[44] Karanasos A Ligthart JM Witberg KT Regar E. Calcified nodules: an underrated mechanism of coronary thrombosis? JACC Cardiovasc Imaging. (2012) 5:1071–2. doi: 10.1016/j.jcmg.2012.04.010 23058076

[45] Li ZY Howarth S Tang T Graves M J U-K-I Gillard JH. Does calcium deposition play a role in the stability of atheroma? Location may be the key. Cerebrovasc Dis. (2007) 24:452–9. doi: 10.1159/000108436 17878727

[46] Lin R Chen S Liu G Xue Y Zhao X. Association between carotid atherosclerotic plaque calcification and intraplaque hemorrhage: a magnetic resonance imaging study. Arterioscler Thromb Vasc Biol. (2017) 37:1228–33. doi: 10.1161/atvbaha.116.308360 28450297

[47] Eisenmenger LB Aldred BW Kim SE Stoddard GJ De Havenon A Treiman GS. Prediction of carotid Intraplaque hemorrhage using adventitial calcification and plaque thickness on Cta. AJNR Am J Neuroradiol. (2016) 37:1496–503. doi: 10.3174/ajnr.A4765 27102316 PMC7960279

[48] Xu X Ju H Cai J Cai Y Wang X Wang Q. High-resolution Mr study of the relationship between superficial calcification and the stability of carotid atherosclerotic plaque. Int J Cardiovasc Imaging. (2010) 26:143–50. doi: 10.1007/s10554-009-9578-3 20052617

[49] Gogela SL Gozal YM Zhang B Tomsick TA Ringer AJ Broderick JP. Severe carotid stenosis and delay of reperfusion in endovascular stroke treatment: an interventional Management of Stroke-iii study. J Neurosurg. (2018) 128:94–9. doi: 10.3171/2016.9.Jns161044 28156253

[50] Huo YC Li Q Zhang WY Zou N Li R Huang SY. Total small vessel disease burden predicts functional outcome in patients with acute ischemic stroke. Front Neurol. (2019) 10:808. doi: 10.3389/fneur.2019.00808 31447754 PMC6691043

[51] Wei C Shen T Tang X Gao Y Yu X Chen X. Cerebral small vessel disease combined with cerebral collaterals to predict the prognosis of patients with acute large artery atherosclerotic stroke. Front Neurol. (2022) 13:969637. doi: 10.3389/fneur.2022.969637 36034278 PMC9403757

[52] Sharifzadeh B Kalbasi R Jahangiri M Toghraie D Karimipour A. Computer modeling of pulsatile blood flow in elastic artery using a software program for application in biomedical engineering. Comput Methods Prog Biomed. (2020) 192:105442. doi: 10.1016/j.cmpb.2020.105442 32192998

[53] Du J Xu Q. Neuroimaging studies on cognitive impairment due to cerebral small vessel disease. Stroke Vasc Neurol. (2019) 4:99–101. doi: 10.1136/svn-2018-000209 31338220 PMC6613873

