# Supplementary figures and images for: Association between carotid plaque calcification and clinical outcomes of symptomatic cerebral small vessel disease
Source: Front Neurol. 2025 Aug 14;16:1628353. doi: 10.3389/fneur.2025.1628353 (PMC12391090; doi:10.3389/fneur.2025.1628353)

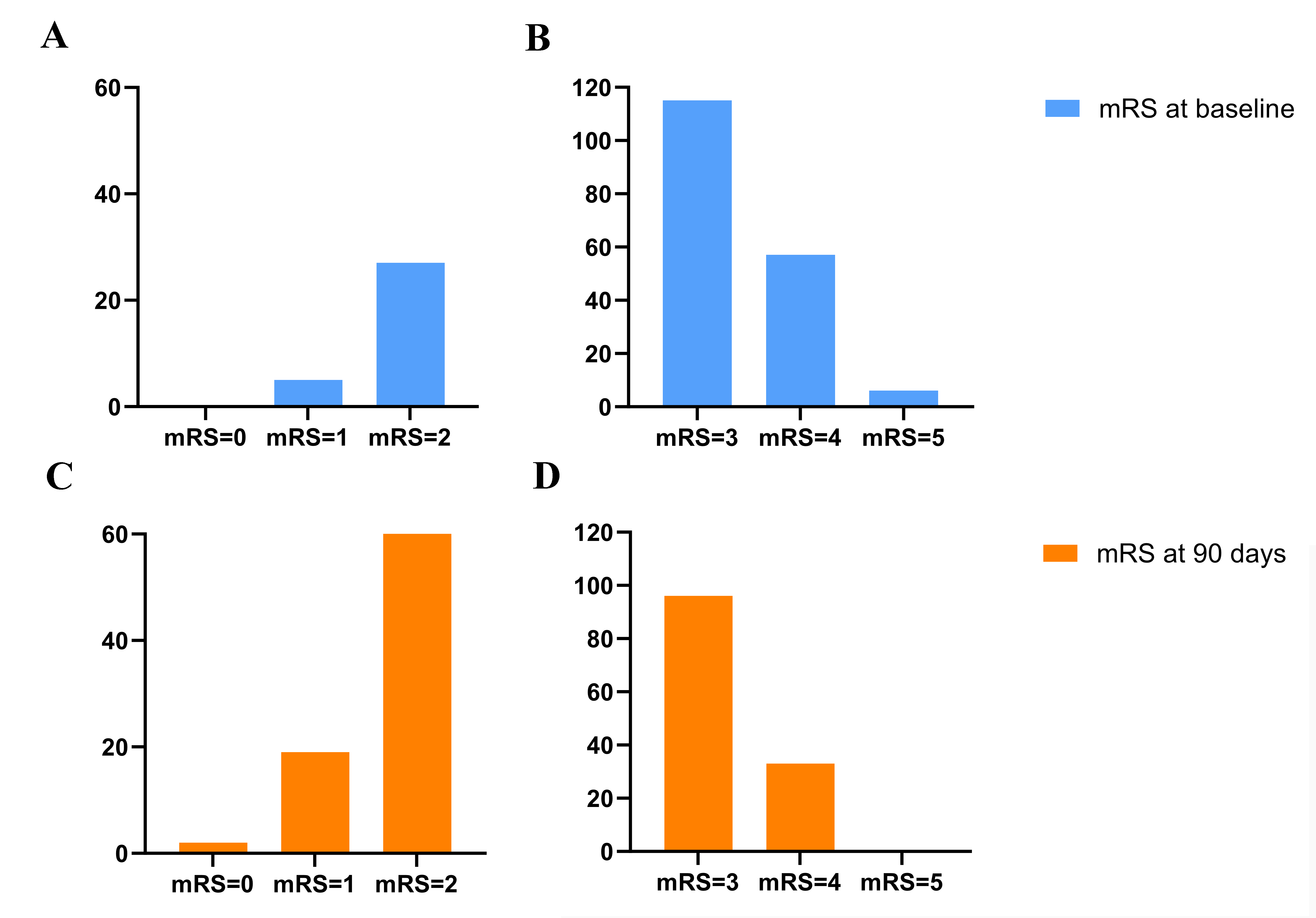

Supplement: SUPPLEMENTARY FIGURE S1 — The distribution of mRS scores at baseline and at 90 days. mRS, 640 modified Rankin scale. [file Image_1.tif]

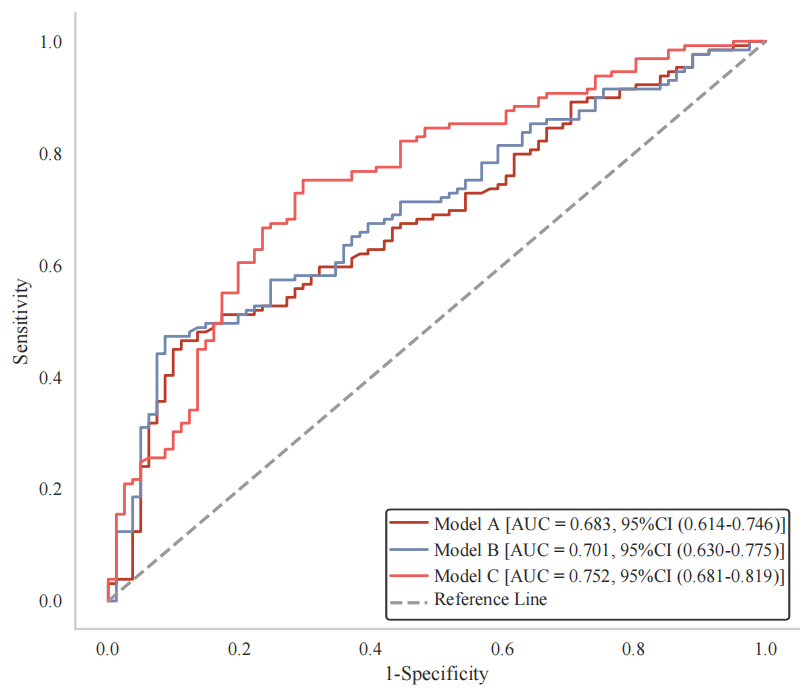

Supplement: SUPPLEMENTARY FIGURE S2 — ROC curves for bootstrap internal validation. AUC, areas under the curve; CI, confidence interval; ROC, receiver operating characteristics. [file Image_2.tif]

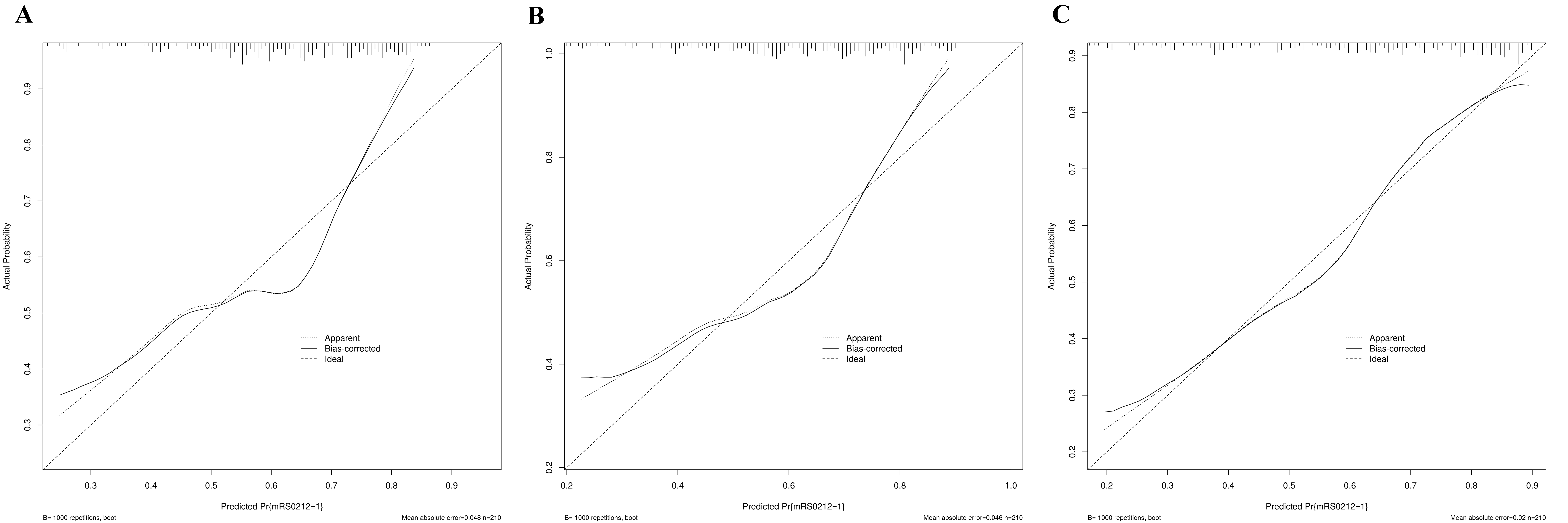

Supplement: SUPPLEMENTARY FIGURE S3 — Calibration curves for multiple models. (A–C) presented calibration curves of Model A–C respectively. [file Image_3.tif]

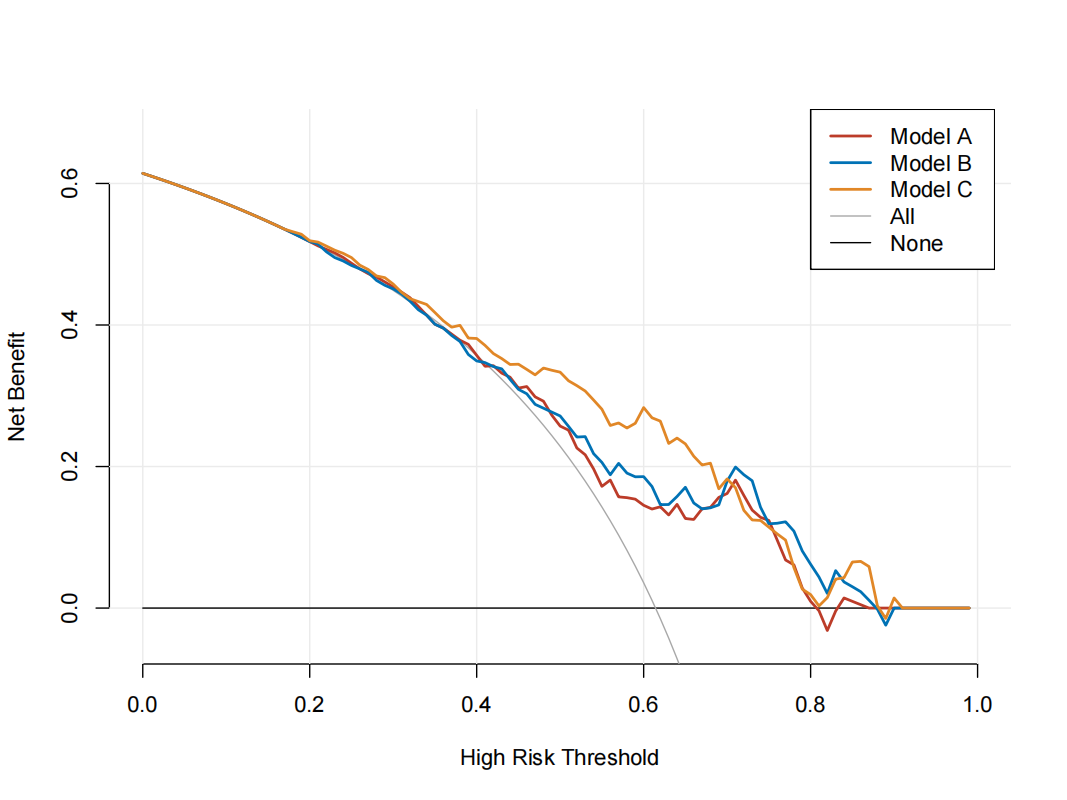

Supplement: SUPPLEMENTARY FIGURE S4 — Decision curves for multiple models. [file Image_4.tif]
