# Supplementary material for: Association between carotid plaque calcification and clinical outcomes of symptomatic cerebral small vessel disease
Source: Front Neurol. 2025 Aug 14;16:1628353. doi: 10.3389/fneur.2025.1628353 (PMC12391090; doi:10.3389/fneur.2025.1628353)
Supplement: Supplementary file 5 [file Table_1.docx]

| Varibales | Univariate  OR (95% CI) | *p* value |
| --- | --- | --- |
| Presence | 3.647 (1.598-8.321) | 0.002^**^ |
| Age*Presence | 0.741 (0.039-14.205) | 0.842 |
| Sex*Presence | 0.291 (0.044-1.905) | 0.198 |
| Hypertension*Presence | 4.909 (0.870-27.686) | 0.071 |
| Diabetes mellitus*Presence | 0.237 (0.017-3.256) | 0.281 |
| Hyperlipidemia*Presence | 0.551 (0.102-2.986) | 0.490 |
| Coronary heart disease*Presence | 1.058 (0.051-22.143) | 0.971 |
| Smoking history*Presence | 1.987 (0.342-11.557) | 0.445 |
| Drinking history*Presence | 1.925 (0.339-10.938) | 0.460 |
| Location |  |  |
| Surface | 2.648 (1.481-4.735) | 0.001^**^ |
| Age*Surface | 0.542 (0.165-1.784) | 0.314 |
| Sex*Surface | 0.449 (0.114-1.770) | 0.252 |
| Hypertension*Surface | 1.587 (0.431-5.843) | 0.487 |
| Diabetes mellitus*Surface | 0.568 (0.141-2.290) | 0.427 |
| Hyperlipidemia*Surface | 1.319 (0.402-4.330) | 0.648 |
| Coronary heart disease*Surface | 0.191 (0.027-1.337) | 0.095 |
| Smoking history*Surface | 0.668 (0.270-1.656) | 0.384 |
| Drinking history*Surface | 1.865 (0.858-4.056) | 0.116 |
| Deep/mixed | 0.710 (0.371-1.357) | 0.300 |
| Number |  |  |
| Single | 0.610 (0.294-1.268) | 0.186 |
| Multiple | 2.886 (1.574-5.290) | 0.001^**^ |
| Age*Multiple | 3.034 (0.662-13.920) | 0.153 |
| Sex*Multiple | 0.535 (0.135-2.120) | 0.373 |
| Hypertension*Multiple | 1.405 (0.369-5.351) | 0.618 |
| Diabetes mellitus*Multiple | 0.502 (0.123-2.049) | 0.337 |
| Hyperlipidemia*Multiple | 0.664 (0.194-2.276) | 0.515 |
| Coronary heart disease*Multiple | 1.872 (0.871-4.021) | 0.108 |
| Smoking history*Multiple | 0.704 (0.203-2.447) | 0.581 |
| Drinking history*Multiple | 0.591 (0.175-1.992) | 0.396 |
| Size |  |  |
| Thin | 0.584 (0.321-1.065) | 0.079 |
| Thick/mixed | 3.062 (1.721-5.451) | <0.001^***^ |
| Age*Thick/mixed | 1.052 (0.988-1.121) | 0.115 |
| Sex*Thick/mixed | 1.564 (0.404-6.062) | 0.517 |
| Hypertension*Thick/mixed | 2.188 (0.593-8.075) | 0.240 |
| Diabetes mellitus*Thick/mixed | 0.312 (0.088-1.112) | 0.073 |

**Table S1** **Logisitic regression of interaction terms between covariates and calcification features.**

| Varibales | Univariate  OR (95% CI) | *p* value |
| --- | --- | --- |
| Hyperlipidemia*Thick/mixed | 1.362 (0.422-4.391) | 0.605 |
| Coronary heart disease*Thick/mixed | 2.376 (0.340-16.590) | 0.383 |
| Smoking history*Thick/mixed | 0.845 (0.258-2.760) | 0.780 |
| Drinking history*Thick/mixed | 0.655 (0.206-2.082) | 0.473 |
| Rim sign | 2.185 (0.583-8.191) | 0.246 |

**Continued Table S1**

^**^ *p* < 0.01, ^***^ *p* < 0.001.

Abbrerations: OR, odds ratio; CI, confidence interval.
